# Turkish adaptation of the Neonatal Eating Assessment Tool–Bottle-Feeding in preterm infants discharged to home

**DOI:** 10.55730/1300-0144.5832

**Published:** 2024-05-23

**Authors:** Burcu AYKANAT GİRGİN, Duygu GÖZEN, Sabiha ÇAĞLAYAN, Britt PADOS

**Affiliations:** 1 Department of Pediatric Nursing, Hamidiye Faculty of Nursing, Health Sciences University, İstanbul, Turkiye; 2 Department of Pediatric Nursing, Faculty of Nursing, Koç University, İstanbul, Turkiye; 3 Department of Pediatric Nursing, Medipol Global International Health Services, İstanbul, Turkiye; 4 Department of Pediatric Nursing, Infant Feeding Care, Wellesley, USA

**Keywords:** Bottle feeding, infant, patient-reported outcome measures, premature, psychometrics

## Abstract

**Background/aim:**

Preterm infants often continue to have feeding difficulties after hospital discharge. Parental use of assessment tools and collaboration with health professionals are important for the early diagnosis of postdischarge feeding difficulties. This methodological study examined the validity and reliability of the Turkish version of the Neonatal Eating Assessment Tool (NeoEAT)–Bottle-feeding in postdischarge preterm infants in Türkiye.

**Materials and methods:**

A Turkish version of the NeoEAT–Bottle-feeding was developed and applied to 321 mothers of preterm infants younger than 7 months of corrected age between August 2021 and December 2022. Cronbach’s alpha, exploratory factor, confirmatory factor, item-total correlation, test-retest, and known-groups validity analyses were performed.

**Results:**

The Turkish NeoEAT–Bottle-feeding has 60 items in five factors explaining 55.785% of the total variance. Exploratory factor analysis indicated that the item factor loading ranged from 0.320 to 0.792. The known-group validity analysis confirmed that preterm infants with diagnosed feeding problems had higher total and subscale scores than those without (p = 0.001). The Cronbach’s alpha (α) of the entire scale was 0.96. The item-total correlation coefficients were between 0.31 and 0.77 (p = 0.001). There was excellent agreement between test values and retest values obtained after a 2-week interval (intraclass correlation coefficient: 0.930–1.000).

**Conclusion:**

The Turkish NeoEAT–Bottle-feeding was shown to be a reliable and valid parent-reported assessment tool for oral feeding skills and difficulties after neonatal intensive care unit discharge in bottle-fed preterm infants younger than 7 months of corrected age. Healthcare professionals can use this assessment tool during the initial evaluation of risk factors contributing to problematic feeding and to determine the effectiveness of planned interventions in preterm infants.

## 1. Introduction

The development of oral feeding skills starts in the intrauterine period through the orchestration of various physiological and biological mechanisms [1,2] and continues postnatally with intra- and intersystemic organization of the neurological, gastrointestinal, cardiorespiratory, and oral-motor systems [3,4]. Preterm infants have problems achieving this intersystemic organization because of their anatomical and physiological immaturity [1,5–7], resulting in poor oral-motor reflexes, a lack of suck-swallow-breath coordination, inability to maintain wakefulness, and physiological instability [1,8–13]. These issues lead to the maladaptive feeding behaviors frequently seen in preterm infants, such as refusal to feed, gagging and coughing during feeding, fussiness, and inadequate food intake [6,14–17].

In many neonatal intensive care units (NICUs), including our own, preterm infants are not discharged until they attain adequate feeding skills [18,19]. However, feeding problems are often observed in the NICU and persist after discharge [5,20,21]. Feeding difficulties involve the infant being unable or unwilling to safely eat enough to maintain hydration and support appropriate growth and development [6]. Preterm infants with postdischarge feeding difficulties may have delayed speech, difficulty transitioning to solid food in the first year of life, compromised growth and development, and impaired parent-infant communication [6,7,9,20–22]. The early diagnosis of feeding difficulties is important to ensure adequate nutrition in the first year of life, support brain development, and prevent developmental delays and long-term feeding problems [23]. Valid and reliable assessment tools are needed to objectively evaluate the feeding skills and behaviors of young infants to allow infants with feeding problems to be identified early, referred for specialist support, and monitored for responses to interventions [15,24]. While breastfeeding is considered optimal nutrition for preterm infants [25], they are given expressed breast milk or formula using alternative methods, most commonly by bottle, until they can be breastfed [25,26].

Various assessment tools have been shown to be valid and reliable in evaluating oral feeding readiness, skills, and difficulties in bottle-fed preterm infants. These assessment tools are administered by clinicians in the NICU [4,27,28]. However, as feeding difficulties frequently persist after discharge in preterm infants, measurement tools completed by parents are important for early diagnosis [15,24]. As opposed to feeding assessments performed at a single time point in the clinic, evaluation by parents who regularly feed their infants and observe their behaviors during each feeding will both increase the parents’ awareness of their infants’ feeding difficulties as well as facilitate the early correction of these problems through clinician-parent cooperation [6,15,24]. For these purposes, the Neonatal Eating Assessment Tool (NeoEAT)–Bottle-feeding was the first measurement tool developed to assess the feeding problems and skills of preterm and term infants based on parental report [15].

In this methodological study, the Turkish translation and adaption of the NeoEAT–Bottle-feeding were performed and its psychometric properties in preterm infants discharged from the NICU in Türkiye were investigated to meet the need for a valid and reliable tool with which to assess parent-reported feeding status and difficulties in preterm infants after discharge in our country.

## 2. Materials and methods

### 2.1. Sample size estimation

This scale adaptation study was conducted between August 2021 and December 2022 with 321 preterm babies and their mothers, who were discharged from the level-three NICU of a private university hospital in İstanbul, Türkiye. Based on the recommended five to 10 times the number of items in the assessment tool [29–31] or 100–500 [32,33] as a good sample size for validity and reliability studies, 321 preterm infants and mothers meeting the study selection criteria were included. The study sample was recruited by random sampling.

Selection criteria for the preterm infants were: 1) gestational age at birth of 26 to 36 + 6 weeks, 2) discharge from the NICU with full oral feeding at least 1 week earlier, 3) corrected age of less than 7 months at the time of study inclusion, and 4) bottle-feeding for the last 7 days before study inclusion. Preterm infants who were fed by any method other than a bottle in the last 7 days were excluded. Selection criteria for the mothers were: 1) age of 18 years or older, 2) being the primary caretaker of the infant, 3) being literate in Turkish, 4) having access to the internet, and 5) volunteering to participate and signing the parental consent form. Mothers with any cognitive, speech, or hearing impairments were excluded.

### 2.2. Ethical considerations

Permission to translate the instrument from English to Turkish was obtained from the developer of the NeoEAT–Bottle-feeding scale via email [15]. In addition, institutional review board approval (24.06.2021/734) and institutional permission were obtained. The mothers were informed about the aim of the study twice, once on the day their infants were discharged from the NICU and again immediately before data collection, and their written consent was obtained.

### 2.3. Instruments

#### 2.3.1. Infant and mother information form

This researcher-created form included eight questions (five open-ended and three multiple choice) about relevant sociodemographic information about the infant (sex, gestational age at birth, birth weight, corrected age at the time of the study, medical diagnoses in the NICU) and mother (age, education level, and financial status) [5,14,15].

#### 2.3.2. Feeding follow-up form

This form collected information about the proportion of food taken by the infant, whether the infant was diagnosed with a feeding impairment after discharge, and if so, the diagnosis. Diagnosis of feeding impairment was made by an independent neonatologist or pediatric gastroenterologist not associated with this research study. Diagnoses were based on their clinical evaluation. On the feeding follow-up form, the mothers were also asked to indicate what proportion of the recommended amount of food was taken by mouth by their infant with three response options: all, more than half, or about half.

#### 2.3.3. NeoEAT–Bottle-feeding

This tool was developed through the collaboration of healthcare professionals and parents to assess the postdischarge feeding skills and problems of preterm and term infants younger than 7 months of corrected age. Its purpose is to identify feeding problems and symptoms early to enable specialized support, foster cooperation between parents and specialists to maintain optimal nutrition, and avoid the long-term effects of feeding disorders [34]. Solid foods are recommended to be introduced at 6 months by the American Academy of Pediatrics but represent a very small proportion of the diet in the first month of transition [35]; thus, it was determined that this tool could be used before the age of 7 months [34]. The original English version of the NeoEAT–Bottle-feeding consists of 64 items organized in five subscales: Infant Regulation (items 1–13), Energy & Physiologic Stability (items 14–25), Gastrointestinal Tract Function (items 26–53), Sensory Responsiveness (items 54–60), and Compelling Symptoms of Problematic Feeding (items 61–64) [15].

The Infant Regulation subscale includes statements related to the infant’s ability to self-regulate, such as “my baby opens mouth to accept the bottle” and “my baby roots when hungry–for example, sucks on fist, smacks lips, looks for bottle”. The Energy & Physiologic Stability subscale consists of statements related to the infant’s ability to maintain energy for feeding and physiologic stability, such as “my baby gets exhausted during eating and is not able to finish” and “my baby can only suck a few times before needing to take a break”. The Gastrointestinal Tract Function subscale contains statements about gastrointestinal functioning, such as “my baby throws up during feeding” and “my baby gags on the bottle nipple”. The Sensory Responsiveness subscale includes items related to the infant’s responses to the sensory experiences of feeding, such as “my baby will only eat from a specific kind of bottle/nipple” and “my baby refuses the bottle before having eaten enough–for example, turns head, pushes bottle away, pushes nipple out of mouth with tongue”. Finally, the Compelling Symptoms of Problematic Feeding subscale consists of items that are related to highly concerning symptoms, such as “my baby needs tube feedings”, “my baby gets pale or blue around lips when eating”, “my baby has blood or mucous in stool”, and “my baby has milk come out of the nose when eating”.

Based on the infant’s feeding behavior after discharge, the mother chooses a response reflecting the frequency of the behavior: never, almost never, sometimes, often, almost always, or always. Most items are scored with never receiving a score of 0 and always receiving a score of 5; however, the Infant Regulation subscale is reverse scored, with never receiving a score of 5 and always receiving a score of 0 [15]. A sum score is determined for each subscale and the total NeoEAT–Bottle-feeding score ranges from 0 to 320 points, with higher scores reflecting more symptoms of problematic feeding [15]. For the original NeoEAT–Bottle-feeding, Cronbach’s α coefficient was 0.92, and total scores correlated strongly in test-retest reliability analysis with an interval of 2 weeks (r = 0.90, p < 0.001). The five subscales explained 43.23% of the variance in the total score, item loadings were acceptable (0.31–0.87), and the known-groups validity analysis showed that typically feeding infants had lower scores than those with diagnosed feeding problems [15].

### 2.4. Procedure

The NeoEAT–Bottle-feeding was translated, back-translated, and evaluated by expert panel review and a pilot test to ensure cross-cultural consistency and content validity before data collection based on Consensus-Based Standards for the Selection of Health Measurement Instruments and current guidelines [36,37].

#### 2.4.1. Forward and back translation

The NeoEAT–Bottle-feeding was independently translated into Turkish by two native Turkish speakers with English fluency and cultural familiarity. The translations were compared, and a single draft was created by selecting the most appropriate expressions from each. A Turkish language expert revised the draft, which was then translated back into English by two native English-speaking translators knowledgeable about health terminology and Turkish language and culture who were not previously involved in the forward translation. Semantic concordance between these versions and the original was evaluated.

#### 2.4.2. Expert opinion

A panel of eight experts comprising six faculty members, a neonatologist, and a neonatal nurse [30,32,38] were given the original and Turkish NeoEAT–Bottle-feeding and asked to assess each item in the Turkish version on a scale of 1 (very appropriate), 2 (appropriate), 3 (requires minor revision), and 4 (requires major revision) [39]. Using the Davis technique, content validity index (CVI) values were calculated as described previously for the items and scale [39,40]. With this technique a CVI >0.80 indicated content validity [39]. After revision based on the expert panel feedback, a pilot test was conducted with the final version [32].

#### 2.4.3. Pilot testing

Twenty mothers who agreed to participate during study enrollment but were not included in the sample participated in the pilot test [31]. Based on their feedback, the scale items were adequately comprehensible. Therefore, this final Turkish version of the instrument was used to collect data from the study sample.

### 2.5. Data collection

Data collection consisted of two stages. In the first stage, the researchers met with eligible mothers of eligible preterm infants on the day of NICU discharge and informed them about the aim and instruments used in the study and told them they would be contacted after discharge for the evaluation their infant’s feeding skills and problems. The mothers’ verbal and written consent were obtained. The infant section of the descriptive information form was completed via a record review, except for the corrected age of the infant at the time of the study. The mothers were asked to complete the mother section of the information form.

In the second stage, the mothers of infants who had been discharged at least 1 week earlier and were less than 7 months of corrected age were contacted by phone and asked to complete the feeding follow-up form and the Turkish NeoEAT–Bottle-feeding online. These were sent to the mothers’ phones and took an estimated 15–20 min for them to complete. Since this stage of the study was conducted via online survey, it was necessary for the mothers to have internet access to complete the data collection. For the test-retest analysis of invariance over time, the Turkish version of the NeoEAT–Bottle-feeding was completed again by 30 mothers 2 weeks after the first assessment [39].

### 2.6. Data analysis

IBM SPSS Statistics for Windows 22.0 and Amos (IBM Corp, Armonk, NY) were used for the statistical analyses. Sociodemographic data were expressed as the number, percentage, mean, and range (minimum–maximum).

The Turkish NeoEAT–Bottle-feeding was evaluated for validity using content, construct, and known-groups analyses. For content validity, CVI values were determined after the expert panel review [39]. Construct validity was ascertained through exploratory factor analysis (EFA) and confirmatory factor analysis (CFA). Before the EFA was performed, the adequacy of the data was assessed using Bartlett’s test of sphericity (p < 0.05 was sought) and the Kaiser–Meyer–Olkin (KMO) test (values between 0.50 and 1 were sought) [32,33,39]. After establishing that the data set was suitable, the principal components method with varimax rotation was used for the EFA. To verify that the Turkish NeoEAT–Bottle-feeding was structurally consistent with the original, CFA was performed, and a series of model fit indices were examined [31,41].

Known-group validity analysis was performed by comparing the total and subscale scores between preterm infants with and without a diagnosed feeding disorder with the hypothesis that scores would be higher in infants diagnosed with feeding disorders. Comparisons between the two known groups were made using Student’s t and Mann–Whitney U tests [15]. Quantitative data were tested for normal distribution using the Shapiro–Wilk test. Student’s t test was used for two-group comparisons of normally distributed quantitative variables and the Mann–Whitney U test was used for nonnormally distributed quantitative variables [31].

The reliability of the Turkish NeoEAT–Bottle-feeding was assessed with Cronbach’s alpha (α), item-total correlation (Pearson correlation), and test-retest (intraclass coefficient correlation) analyses, with p < 0.05 regarded as statistically significant. Diagnostic screening tests (specificity, sensitivity) in receiver operating characteristic (ROC) curve analysis were used to determine the optimum cut-off value for the Turkish NeoEAT–Bottle-feeding total score [32].

## 3. Results

### 3.1. Sample characteristics

A total of 321 preterm infants and their mothers were included in the sample. The mean gestational age was 33.51 ± 2.95 weeks, birth weight was 1562 ± 457.5 g, and 55.1% (n = 177) of the infants were male. The most common diagnoses during their NICU stay were transient tachypnea of the newborn (49.2%, n = 158), respiratory distress syndrome (26.8%, n = 86), and congenital heart diseases (11.5%, n = 37). The corrected mean age of the preterm infants at the time of enrollment in the study was 11.18 ± 6.94 weeks, 75.1% (n = 241) ate all the food given, 6.5% (n = 21) were diagnosed with feeding disorders, and the most common disorder was gastroesophageal reflux (3.1%, n = 10) (Table 1).

According to their corrected age at the time of the study, 49.5% (n = 159) of the preterm infants were 0–2 months old, 19.7% (n = 63) were between 2 months 1 day and 4 months old, and 30.8% (n = 99) were between 4 months 1 day and 6 months old. When the sex of the preterm infants was examined according to their corrected age at the time of the study, males accounted for 52.8% (n = 84) of infants at a corrected age of 0 to 2 months, 63.5% (n = 40) of those at a corrected age between 2 months 1 day and 4 months, and 53.5% (n = 53) of those at a corrected age between 4 months 1 day and 6 months (Table 2).

The mothers had a mean age of 31.0 ± 6.0 (range: 18–47) years, most were university (50.5%, n = 162) or high school (48.3%, n = 155) graduates, and their income level was equal to their expenses in 54.8% (n = 176) and less than their expenses in 41.1% (n = 132).

### 3.2. Validity analysis

#### 3.2.1. Content validity

As a result of the expert panel review, the item-level CVI values were 0.80–1.00 and the scale-level CVI was 0.96.

#### 3.2.2. Construct validity

The original NeoEAT–Bottle-feeding consists of 64 items. In the EFA, four items were identified and removed in the original Gastrointestinal Tract Function subscale that loaded similarly on multiple factors and had factor loadings below 0.30 (item 36: my baby is very gassy; item 40: my baby needs to be burped more than once before the end of feeding; item 42: my baby turns red in face, may cry with stooling/pooping; and item 53: my baby has hard stools/poop). In the original, these are items 11, 15, 17, and 28 of the Gastrointestinal Tract Function Subscales. The remaining 60 items were included in the EFA. Bartlett’s χ^2^ test was significant (p = 0.001) and the KMO coefficient was 0.931. EFA demonstrated that the 60 items in the Turkish version conformed to the same 5-factor structure as the original (factor 1: Infant Regulation, 13 items [items 1–13]; factor 2: Energy & Physiologic Stability, 12 items [items 14–25]; factor 3: Gastrointestinal Tract Function, 24 items [items 26–49]; factor 4: Sensory Responsiveness, 7 items [items 50–56]; and factor 5: Compelling Symptoms of Problematic Feeding, 4 items [items 57–60]) which explained 55.8% of the total variance of the scale. It was determined that 17.6% of the total variance was explained by the Infant Regulation subscale, 15.4% by the Energy & Physiologic Stability subscale, 9.4% by the Gastrointestinal Tract Function subscale, 8.4% by the Sensory Responsiveness subscale, and 4.9% by the Compelling Symptoms of Problematic Feeding subscale. Item factor loadings ranged from 0.32 to 0.79. Item factor loadings in the subscales were 0.37–0.69 for Infant Regulation, 0.32–0.78 for Energy & Physiologic Stability, 0.37–0.79 for Gastrointestinal Tract Function, 0.38–0.64 for Sensory Responsiveness, and 0.32–0.70 for Compelling Symptoms of Problematic Feeding (Table 3).

**Figure 1:**
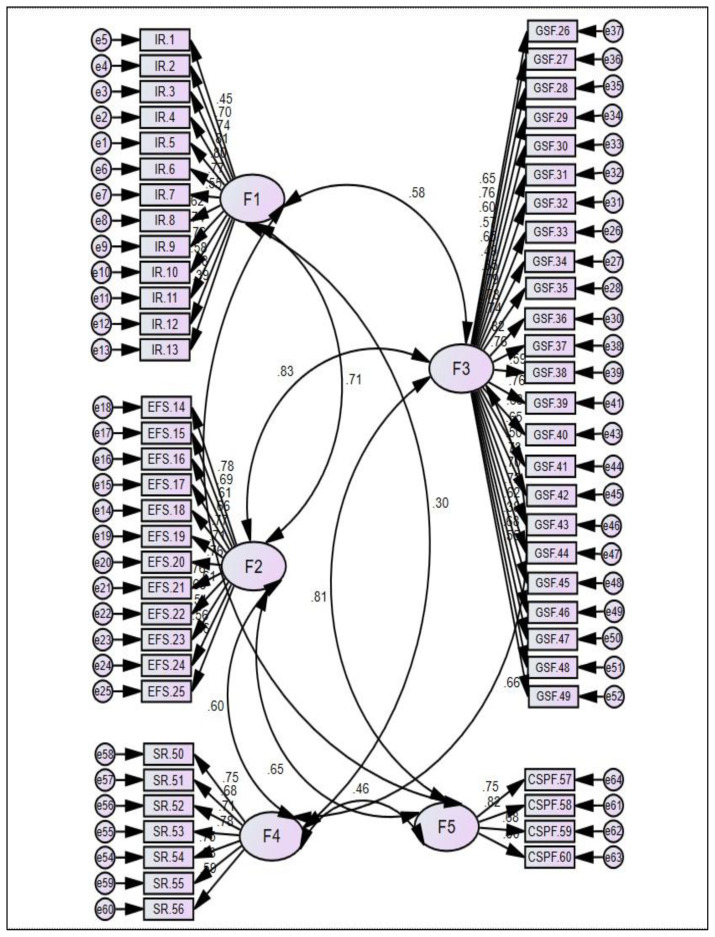
Factor structure of the Turkish version of the NeoEAT–Bottle-feeding For all factor loadings p < 0.001. * IR: Infant Regulation; EFS: Energy & Physiologic Stability; GSF: Gastrointestinal Tract Function; SR: Sensory Responsiveness; CSPF: Compelling Symptoms of Problematic Feeding.

As in the EFA, CFA was performed with 60 items after removing the same items (items 11, 15, 17, and 28 of the Gastrointestinal Tract Function subscale) with factor loadings below 0.30. Item factor loadings in the subscales were 0.39–0.80 for Infant Regulation, 0.50–0.78 for Energy & Physiologic Stability, 0.38–0.81 for Gastrointestinal Tract Function, 0.58–0.77 for Sensory Responsiveness, and 0.67–0.90 for Compelling Symptoms of Problematic Feeding (Figure 1). Model fit indices obtained in the CFA were as follows: chi-squared/degrees of freedom (χ^2^/df) ratio = 3.98, root mean square error of approximation (RMSEA) = 0.07, comparative fit index = 0.94, normal fit index = 0.96, incremental fit index = 0.95, goodness of fit index = 0.93, and relative fit index = 0.93 (Table 4).

#### 3.2.3. Known-groups validity, cut-off value, sensitivity, and specificity

**Figure 2:**
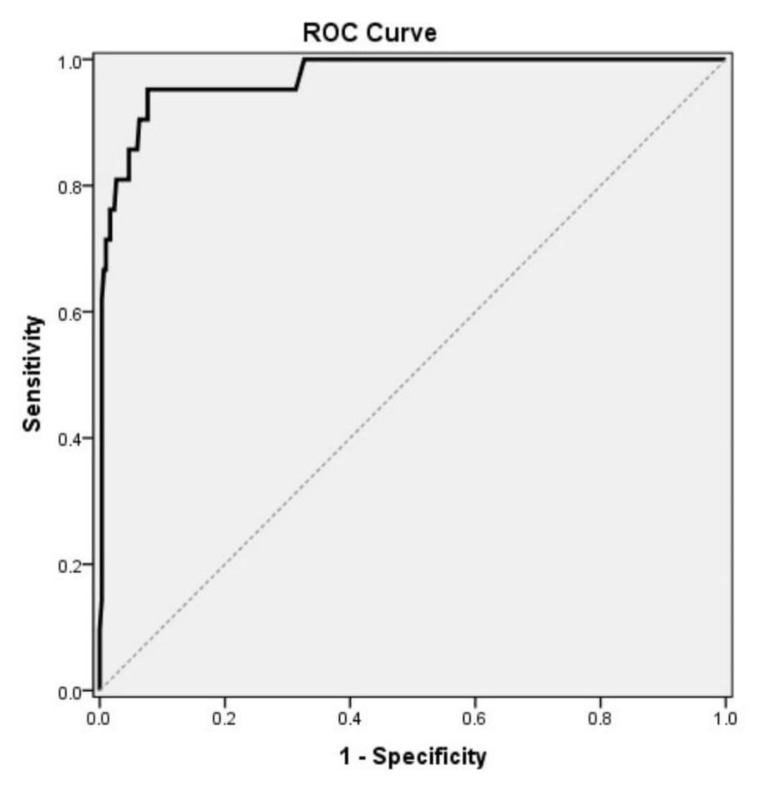
Determination of the cut-off value according to the ROC analysis.

The subscale and total scores on the Turkish NeoEAT–Bottle-feeding were significantly higher in the group of preterm infants with diagnosed feeding disorders when compared with those without (Table 5, p = 0.001). Based on this significance, a cut-off value was calculated for the Turkish NeoEAT–Bottle-feeding total score. According to diagnosed feeding disorders, the optimum cut-off value was 97. At this cut-off value, the NeoEAT–Bottle-feeding total score had 95.2% sensitivity, 92.3% specificity, a positive predictive value of 46.5%, a negative predictive value of 99.6%, and 92.5% accuracy (Table 6, Figure 2). The area under the ROC curve (AUC) was 97.2% with 1.6% standard error.

### 3.3. Reliability analysis

The Cronbach’s α was 0.96 for the entire Turkish NeoEAT–Bottle-feeding and was 0.90, 0.91, 0.94, 0.87, and 0.87 for the Infant Regulation, Energy & Physiologic Stability, Gastrointestinal Tract Function, Sensory Responsiveness, and Compelling Symptoms of Problematic Feeding subscales, respectively.

The item-total correlation coefficients were below 0.30 for items 11 (r = 0.28), 15 (r = 0.26), 17 (r = 0.21), and 28 (r = 0.21) of the Gastrointestinal Tract Function subscales, which were removed during the EFA. For the remaining 60 items, the item-total correlation coefficients were between 0.31 and 0.77 (p = 0.001). The item-total correlation coefficients for the subscales were 0.31–0.68 for Infant Regulation, 0.49–0.76 for Energy & Physiologic Stability, 0.46–0.77 for Gastrointestinal Tract Function, 0.39–0.70 for Sensory Responsiveness, and 0.51–0.70 for Compelling Symptoms of Problematic Feeding.

Thirty parents (9.3% of the total sample) completed the test and retest 2 weeks apart, and the intraclass correlation coefficients were between 0.930 and 1.000 for all the items (p = 0.001), indicating excellent agreement between the 2 measurements.

Based on the results of the validity and reliability analyses, the final Turkish version of the NeoEAT–Bottle-feeding included 60 items in 5 subscales. The total score obtained from the scale varies from 0 to 300.

## 4. Discussion

Described herein was the rigorous process followed to translate and culturally adapt the NeoEAT–Bottle-feeding from English to Turkish. The Turkish version of the NeoEAT–Bottle-feeding has evidence of adequate psychometric properties in infants under 7 months corrected age who were born preterm, including content, construct, and known-groups validity, as well as internal consistency and test-retest reliability.

The item-level and scale-level CVIs in the current study indicated that the items of the Turkish NeoEAT–Bottle-feeding adequately represented the construct being measured and were appropriate for Turkish culture [29,30,39,42]. However, the CVI values for the original NeoEAT–Bottle-feeding scale were not provided in the study of Pados et al. (2018), making a direct comparison challenging [15]. EFA can be performed if the KMO coefficient is greater than 0.50 and Bartlett’s chi-squared test gives a significant result [29,39,43]. In the present study, the results of these tests indicated the sample size and data set were sufficient and suitable for factor analysis [29,43]. Pados et al. reported comparable values for the original version (p *<* 0.001 for Bartlett’s χ^2^, KMO coefficient = 0.905) [15].

The original English version of the NeoEAT–Bottle-feeding consisted of 64 items in five subscales [15]. The Turkish version also consists of five subscales that are consistent with the original in terms of the subscale names and included items. However, four items that were included in the English version were removed because their factor loading values were below 0.30 in the EFA [29,30,42]. The original authors noted that one of these items (My baby has hard stools/poop) also failed to load at 0.30 or greater, but they chose to retain it because they felt it was important clinically and it did not negatively impact the Cronbach’s α [15]. The other three items that were removed had factor loadings of 0.30 or greater in the original study of the English version of the tool [15]; the difference between the two studies could be a result of cultural interpretation of the infant behaviors described in these items. For example, there may be cultural differences in how parents determine whether their baby is “very gassy” or “needs to be burped more than once before the end of a feeding”. The factor loadings of the other 60 items were between 0.32 and 0.79, so these items were retained [30,42]. The factor loadings for the original scale were 0.31–0.87 [15], consistent with the current study. The five-factor structure of the Turkish NeoEAT–Bottle-feeding explained 55.79% of the total variance, which was above the desired threshold of 40% for multifactor scales [30,43] and greater than that reported in the original study of 43.23% [15]. The higher total explained variance in the present study compared to the original study indicates that the concepts in the scale were effectively measured in the Turkish sample [30]. The 5-factor model had item factor loadings above 0.30 for the CFA (Figure 1), χ^2^/df below 5, RMSEA below 0.08, and other fit index values above 0.90 (Table 4). These results demonstrated an acceptable level of fit [32,42,43].

Preterm infants diagnosed with feeding problems received higher NeoEAT–Bottle-feeding subscale and total scores compared to those without in the current study, demonstrating known-groups validity. Consistent with these findings, Pados et al. (2018) found that the total NeoEAT–Bottle-feeding score of healthy, typically feeding term infants was lower than that of infants with feeding problems (103.1 ± 37.9 vs. 66.6 ± 25.6, p < 0.001) [15]. Unlike the present study; however, they observed no significant difference in the infant regulation subscale scores between the 2 groups. They indicated that the lack of statistical significance in the known-groups validation for the infant regulation subscale was likely due to the small sample size in the 6- to 7-month age group [15]. The difference between the two studies in the infant regulation results may also be related to the sample groups. The current study compared preterm infants with and without a diagnosis of feeding disorder, whereas the original study included a mixed sample of healthy, term infants, preterm infants, and infants with other medical diagnoses, comparing scores between infants with and without a parent-reported feeding problem. As a result of the ROC analysis, the optimum cut-off value of the Turkish NeoEAT–Bottle-feeding total score for the diagnosis of feeding problems was 97 (Table 6). Preterm infants with a score of 97 or higher on the Turkish NeoEAT–Bottle-feeding can be evaluated as having a high level of feeding problems or a feeding disorder. This cut-off value had the highest sensitivity and specificity values (95.2% and 92.3%, respectively). Sensitivity refers to the proportion of subjects who have the target condition and have positive test results, while specificity refers to the proportion of subjects who do not have the target condition and have negative test results [44,45]. In ROC curve analysis, an AUC of 0.70–0.80 is acceptable, 0.80–0.90 is very good, and greater than 0.90 is excellent [44–46]. The AUC in the present study was 0.972 (Table 6), indicating that the Turkish NeoEAT–Bottle-feeding also has significant ability to distinguish preterm infants with and without feeding disorder (p = 0.001).

The findings herein related to the reliability of the Turkish NeoEAT–Bottle-feeding were consistent with those of the original study, in which the authors reported a Cronbach’s α of 0.92 [15]. For the Turkish version, the Cronbach’s α was 0.96. Again, the difference is likely a result of the more homogeneous sample of preterm-born infants in the current study, but suggests that the Turkish version has excellent internal consistency reliability. There was also excellent agreement between the two measurements for the test-retest analysis performed with 30 mothers at a 2-week interval in the present study, demonstrating temporal stability reliability [32]. These findings are consistent with those reported for the original version [15].

Like the original English version, the Turkish version of the NeoEAT–Bottle-feeding is a parent-reported assessment of symptoms of problematic feeding intended for infants under 7 months of age that has evidence of adequate psychometric properties for use in clinical practice and research. The NeoEAT–Bottle-feeding provides an objective assessment of bottle-feeding that does not require specialized training, is inexpensive to administer, and utilizes the parent as the expert on the infant’s feeding [15]. In this respect, it differs from the Early Feeding Skills (EFS) assessment tool, which is not suitable for use by parents. The EFS evaluates preterm/term infants based on the observations of trained clinicians while in the NICU [4]. However, feeding problems encountered in preterm infants in the NICU often continue after discharge [5,16,21]. The NeoEAT–Bottle-feeding can be used for cooperative monitoring of preterm/term infants’ feeding status after NICU discharge between parents and neonatal follow-up clinics and primary care clinicians and facilitate the referral of infants at risk of feeding problems to expert support. In this way, it may help to prevent long-term feeding problems in this population of infants who are known to be at high risk [14,15, 47].

This study had a few limitations. First, the research methods required mothers to have access to a phone and the internet. Future studies could offer additional methods of completing the survey to ensure that those without access to a phone or the internet could participate. Second, the cut-off value was calculated for infants of all ages. Future work with larger sample sizes of infants in each age group could determine age-specific reference values. Finally, although the original scale continues to be adapted into different languages, the validity and reliability data for these versions have not yet been published. Therefore, in the discussion section, a comparison could only be made with data pertaining to the original scale.

## 5. Conclusion

The Turkish version of the NeoEAT–Bottle-feeding comprises 60 items in the same five subscales as the original English version. The results demonstrated that the Turkish NeoEAT–Bottle-feeding is a valid and reliable parent-reported measure of symptoms of problematic feeding in bottle-fed preterm infants younger than 7 months of corrected age after NICU discharge. With the validity and reliability of the Turkish version of the NeoEAT–Bottle-feeding established, there will be opportunities for future research into the treatment and management of Turkish infants with feeding difficulties. Those interested in obtaining the Turkish version of the NeoEAT–Bottle-feeding should contact the first author.

## Figures and Tables

**Table 1:** Descriptive and clinical characteristics of the preterm infants and mothers (n = 321)

Characteristics		n	%
Sex	Female	144	44.9
	Male	177	55.1
Gestational age at birth (weeks)	Mean ± SD	33.51 ± 2.95	
	Median (range)	34.7 (24–35.9)	
Birth weight (g)	Mean ± SD	1562 ± 457.5	
	Median (range)	560 – 2660	
Corrected age at the study (weeks)	Mean ± SD	11.18 ± 6.94	
	Median (range)	8.3 (0.1–24.3)	
Medical diagnosis*	Transient tachypnea of the newborn	158	49.2
	Respiratory distress syndrome	86	26.8
	Congenital heart disease (PDA, ASD, VSD, tetralogy of Fallot)	37	11.5
	Pneumonia	21	6.5
	Sepsis	19	5.9
	Hypoglycemia	12	3.7
	Hyperbilirubinemia	10	3.1
	Bronchopulmonary dysplasia	6	1.9
	Necrotizing enterocolitis	4	1.2
	Atresia (rectal, choanal, jejunal)	4	1.2
	Epilepsy	3	0.9
Proportion of food taken	All	241	75.1
	More than half	61	19.0
	About half	19	5.9
Feeding impairment	Yes	21	6.5
	No	300	93.5
Diagnosed feeding impairment (n = 21)	Vomiting	5	1.6
	Gastroesophageal reflux	10	3.1
	Feeding intolerance	6	1.8
Mothers’ age	Mean ± SD	31.00 ± 6.00	
	Median (range)	31 (18–47)	

PDA: Patent ductus arteriosus, ASD: atrial septal defect, VSD: ventricular septal defect,

* one infant had more than one diagnosis.

**Table 2:** Sex of the preterm infants according to their corrected ages (n = 321).

Corrected ages	Female		Male	
	n	%	n	%
0–2 months (n = 159)	75	47.2	84	52.8
>2–4 months (n = 63)	23	36.5	40	63.5
>4–6 months (n = 99)	46	46.5	53	53.5

**Table 3:** EFA results of the Turkish version of the NeoEAT–Bottle-feeding (n= 321).

Items	Item factor loadings				
	Infant Regulation	Energy & Physiologic Stability	Gastrointestinal Tract Function*	Sensory Responsiveness	Compelling Symptoms of Problematic Feeding
Item 1	0.436				
Item 2	0.558				
Item 3	0.461				
Item 4	0.374				
Item 5	0.377				
Item 6	0.450				
Item 7	0.493				
Item 8	0.690				
Item 9	0.519				
Item 10	0.648				
Item 11	0.644				
Item 12	0.640				
Item 13	0.385				
Item 14		0.698			
Item 15		0.622			
Item 16		0.506			
Item 17		0.540			
Item 18		0.613			
Item 19		0.777			
Item 20		0.641			
Item 21		0.724			
Item 22		0.335			
Item 23		0.320			
Item 24		0.434			
Item 25		0.755			
Item 26			0.574		
Item 27			0.496		
Item 28			0.727		
Item 29			0.391		
Item 30			0.632		
Item 31			0.604		
Item 32			0.556		
Item 33			0.570		
Item 34			0.700		
Item 35			0.689		
Item 36			0.703		
Item 37			0.705		
Item 38			0.792		
Item 39			0.533		
Item 40			0.740		
Item 41			0.766		
Item 42			0.753		
Item 43			0.640		
Item 44			0.374		
Item 45			0.628		
Item 46			0.617		
Item 47			0.557		
Item 48			0.727		
Item 49			0.628		
Item 50				0.550	
Item 51				0.383	
Item 52				0.391	
Item 53				0.523	
Item 54				0.557	
Item 55				0.641	
Item 56				0.544	
Item 57					0.463
Item 58					0.697
Item 59					0.324
Item 60					0.515
Explained variance (%)	17.551	15.449	9.405	8.447	4.883
Total explained variance (%)	55.785				

* Four items in the original scale were removed and the Turkish version was renumbered. There were 27 items in the original subscale and 23 items in the Turkish version. The original scale consists of 64 items and the Turkish version consists of 60 items.

**Table 4:** Model fit indices for the CFA (n = 321).

Model	χ^2^	df^a^	χ^2^/df	RMSEA^b^	GFI^c^	CFI^d^	NFI^e^	IFI^f^	RFI^g^
Five-factor model	774.665	252	3.98	0.073	0.93	0.94	0.96	0.95	0.93

a Degrees of freedom,

b root mean square error of approximation,

c goodness of fit index,

d comparative fit index,

e normed fit index,

f incremental fit index, and

g relative fit index.

**Table 5:** Comparison of the NeoEAT–Bottle-feeding scores of the preterm infants according to the presence of a diagnosed feeding disorder (n = 321).

	Diagnosed feeding disorder				p-value
	Yes (n = 21)		No (n = 300)		
	Mean ± SD	Median (range)	Mean ± SD	Median (range)	
Infant Regulation	35.1 ± 7.4	36 (17–46)	18.2 ± 7.2	18 (2–47)	a0.001**
Energy & Physiologic Stability	28.1 ± 9.6	30 (5–40)	12.9 ± 7.5	12 (0–44)	a0.001**
Gastrointestinal Tract Function	53.0 ± 16.5	50 (14–81)	13.9 ± 10.0	12 (0–66)	a0.001**
Sensory Responsiveness	16.7 ± 7.1	20 (2–28)	6.8 ± 6.8	5 (0–29)	b0.001**
Compelling Symptoms of Problematic Feeding	5.4 ± 2.7	5 (2–11)	0.3 ± 0.9	0 (0–10)	b0.001**
Total NeoEAT–Bottle-feeding	145.8 ± 36.9	144 (67–216)	58.6 ± 25.5	54 (9–192)	a0.001**

a Student’s t test,

b Mann–Whitney U test,

** p < 0.01.

**Table 6:** Diagnostic parameters and ROC curve analysis results for the Turkish version of the NeoEAT–Bottle-feeding total score.

	Diagnostic parameter					ROC curve		p-value
	Cut-off point	Sensitivity	Specificity	Positive predictive value	Negative predictive value	AUC*	95% Confidence interval	
NeoEAT–Bottle-feeding total score	≥97	95.24	92.33	46.51	99.64	0.972	0.941–1.000	0.001

* AUC.
